# Volatiles of *Capparis cartilaginea* Decne. from Saudi Arabia

**DOI:** 10.3390/plants11192518

**Published:** 2022-09-26

**Authors:** Bashaer Alsharif, Grace Adebusola Babington, Niko Radulović, Fabio Boylan

**Affiliations:** 1 School of Pharmacy and Pharmaceutical Sciences, Trinity Biomedical Sciences Institute, Trinity College Dublin, D02 PN40 Dublin, Ireland; 2 Department of Pharmacognosy, Faculty of Pharmacy, Umm Al-Qura University, Makkah 21955, Saudi Arabia; 3 Department of Chemistry, Faculty of Sciences and Mathematics, University of Niš, 18000 Niš, Serbia

**Keywords:** *Capparis cartilaginea*, essential oil analysis, volatiles, principal component analysis, agglomerative hierarchical cluster analysis

## Abstract

In this study, GC and GC–MS analysis of the essential oil obtained from the leaves of Saudi Arabian Capparis cartilaginea Decne. (CC) allowed for the identification of 41 constituents, comprising 99.99% of the total oil composition. The major compounds identified were isopropyl isothiocyanate (31.0%), 2-methylbutanenitrile (21.4%), 2-butyl isothiocyanate (18.1%), isobutyronitrile (15.4%), and 3-methylbutanenitrile (8.2%). The chemical composition of the derived oil and 12 additional oils obtained from selected Capparis taxa were compared using multivariate analyses including principal component analysis (PCA) and agglomerative hierarchical cluster analysis (AHC). The results of the statistical analyses of this particular data set pointed out that isopropyl isothiocyanate could be potentially used as a valuable infrageneric chemotaxonomical marker for CC. Moreover, the results distinctly separate CC from other members of its genus on the basis of its components. In addition, environmental and geographical stressors may be implicated in the essential oil profile of plants found within the genus *Capparis.*

## 1. Introduction

The genus *Capparis* is comprised of nearly 400 taxa dispersed in tropical and subtropical regions of the Mediterranean, Middle East, Southwest Asia, and Northern Africa characterised by arid conditions. A member of the Capparaceae family, the genus *Capparis* can be concisely described as evergreen, often climbing trees or shrubs with short, frequently recurved stipular spines [1]. *Capparis cartilaginea* Decne. (CC), a scandent shrub, has been identified as a native plant of North Africa, Western Asia, and spanning up to India and the Arabian Peninsula [2]. Ethnobotanical data delineate the use of CC leaves in tropical Africa as a laxative as well as a remedy to treat eye infections while the roots are utilised to treat skin diseases and ulcers. CC has also been historically used for the treatment of rheumatism, gout, and tuberculosis in countries such as Pakistan and India. Similarly, CC has also been adopted for its diuretic, expectorant, anthelmintic, and emmenagogue properties [3,4,5]. In traditional Arabian medicine, CC is used for the treatment of inflammation, earache, headache, healing of bruises, snakebite, and childbirth [6].

Many phytochemical studies on the genus *Capparis* have shown that different parts of the plant contain terpenoids, flavonoids, alkaloids, glucosinolates, isothiocyanates, sterols, and fatty acid [7]. However, compared to other *Capparis* species, there are few studies conducted on CC. In 1997, four flavonoids were isolated and identified from CC from Egypt, namely, kaempferol 3-rutinoside (nicotiflorin), quercetin 3-rutinoside (rutin), quercetin-7-rutinoside, and quercetin 3-glucoside-7-rhamanoside [8]. In addition, four isothiocyanates were isolated and identified from CC extract using GC and EI/MS techniques. These compounds were butyl isothiocyanate, 6-methylsulphonylhexyl isothiocyanate, 7-methylsulphonylheptyl isothiocyanate, and 5-benzylsulphonyl-4-pentenyl isothiocyanate [9]. CC have reported a range of biological activities including antioxidant, anticancer, antimicrobial, and anti-osteoporotic and larvicidal effects [10,11,12,13,14]. Research has also been conducted to illustrate the hypotensive and spasmolytic activities by whole plant extracts of CC [15]. 

The current body of research analysing the essential oil composition of CC is practically non-existent. Therefore, the aim of this research article is to report the findings of the critical evaluation of CC essential oil composition by means of GC/GC–MS and subsequent comparison to previous studies using multivariate analyses (MVA), namely, agglomerative hierarchical cluster analysis (AHC) and principal component analysis (PCA).

## 2. Results

Hydrodistillation of the air-dried leaves of CC yielded a yellowish-green oil with a sharp pungent scent. A total of 99.99% of peak areas corresponding to 41 components were identified by GC and GC–MS analyses (Table 1). The chromatogram is in the Supplementary Materials (Figure S1).

The oil was dominated by sulphur containing compounds, isothiocyanates, accounting for 50.0% of the analysed oil. This was followed closely by nitriles accounting for 47.4% of the analysed oil. Other classes of compounds identified include monoterpenes (1.9%) and esters (0.3%).

**Figure 1:**
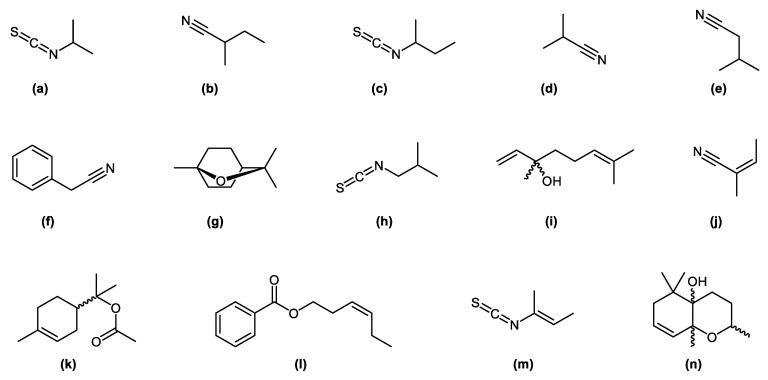
Chemical structure of some of the characteristic constituents from the leaf essential oil of CC.

These compounds have previously been reported as characteristic constituents of some other *Capparis* species [16,17]. Table 1 outlines all constituents identified and their percentage content. The majority of the compounds identified are consistent with other reported *Capparis* species; however, most of the nitrile containing compounds (2-methylbutanenitrile; 3-methylbutanenitrile; isobutyronitrile and 2-methyl-3-butenenitrile) appear to have been identified for the first time in a *Capparis* species (present study) (see Table 1). The chemical structures of some of the characteristic compounds present in the analysed oil are given in Figure 1.

**Figure 2:**
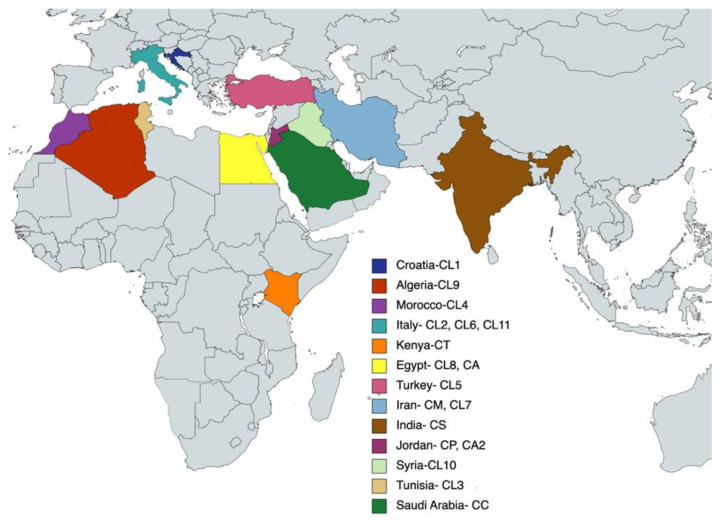
EMEA map showing essential oil samples considered in this study.

The demographics of the essential oils of various *Capparis* species considered for the purpose of this study are listed in Table 2 and Figure 2. Twelve samples in total were analysed using multivariate analyses, i.e., AHC and PCA, to be determine based on their essential oil profiles for potential intergeneric relationships of the taxa. These samples are annotated with an asterisk (*).

**Table 2:** List of essential oil samples used for statistical analysis.

Taxon	Plant Part	Origin	Designation	Reference
*Capparis cartilaginea* Decne. *	Leaves	Saudi Arabia	CC	Present Study
*Capparis spinosa* var. *aegyptiaca* *	Aerial parts	Egypt	CA	[18]
*Capparis spinosa* L. *	Buds and Leaves	Croatia	CL1	[19]
*Capparis tomentosa* *	Leaves and Fruits	Kenya	CT	[20]
*Capparis spinosa* var. *mucronifolia* *	Fruit	Iran	CM	[21]
*Capparis ovata* Desf. var. *palaestina* *	Aerial parts	Jordan	CP	[22]
*Capparis spinosa* L. var. *aravensis* *	Aerial parts	Jordan	CA2	[22]
*Capparis spinosa* L. *	Leaves	Syria	CL10	[23]
*Capparis spinosa* L. *	Leaves	Italy	CL11a	[17]
*Capparis spinosa* L. *	Buds	Italy	CL11b	[17]
*Capparis spinosa* L. *	Flowers	Italy	CL11c	[17]
*Capparis spinosa* L.	Buds	Italy	CL2	[24]
*Capparis spinosa* L.	Seeds	Tunisia	CL3	[25]
*Capparis spinosa* L.	Buds	Morocco	CL4	[26]
*Capparis spinosa* L.	Buds	Turkey	CL5	[27]
*Capparis spinosa* L.	Buds	Italy	CL6	[27]
*Capparis spinosa* L. *	Seeds	Iran	CL7	[27]
*Capparis spinosa* L.	Aerial parts	Egypt	CL8	[28]
*Capparis spinosa* L.	Aerial parts	Algeria	CL9	[29]
*Capparis sepiaria* Linn.	Seeds	India	CS	[30]

* Asterisks mark the samples analysed using multivariate analysis.

**Figure 3:**
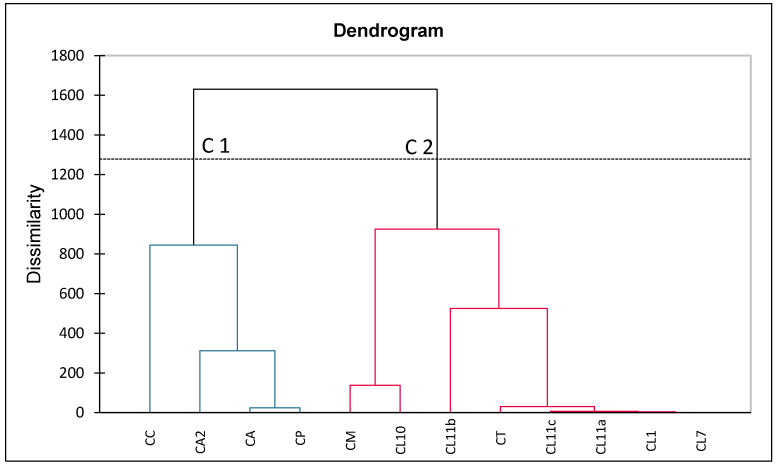
Dendrogram (AHC analysis) representing the chemical composition dissimilarity relationships of 12 essential oil samples (observations) obtained by Euclidian distance dissimilarity using the aggregation criterion of Ward’s method. Two main groups of samples (C1 and C2) were found.

**Figure 4:**
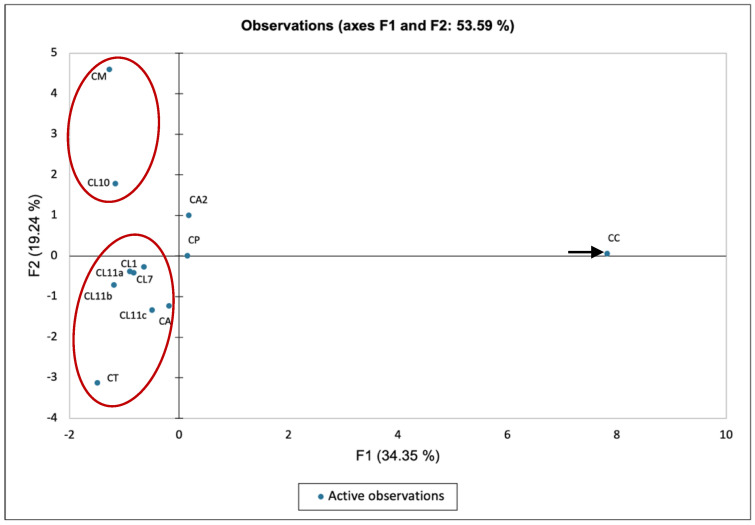
Principal component analysis ordination of 12 oil samples (observations). Axes (F1 and F2 correspond to the first and second principal component responsible. Axis F1 accounts for ca. 34.35% and axis F2 for a further 19.24% of the total variance.

The resultant essential oil analysis of *Capparis* species regardless of plant part or geographical location were considered in this statistical analysis with the only exclusion criteria being complete absence of similarity to CC (present study). Evidently, excluded samples shared no chemical constituents at a quantity of 1% or over with the oil analysed in the present study. Due consideration of the impact of environmental factors and plant organ specification is factored into the interpretation of the MVA results presented in Figure 3 and Figure 4.

## 3. Discussion

**Figure 5:**
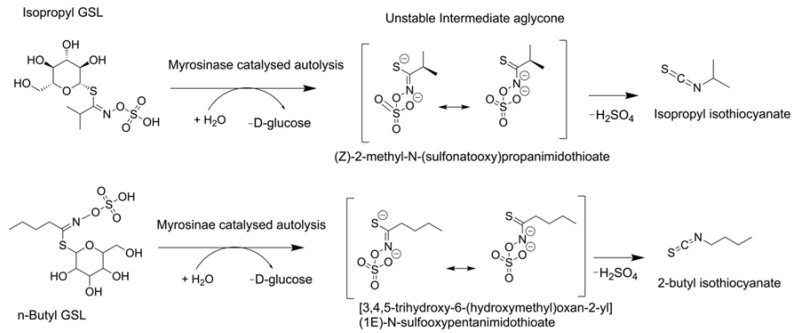
Schematic reaction mechanism showing GSL autolysis, intermediate unstable aglycone and final products.

The major constituents identified were isopropyl isothiocyanate (31.0%), 2-methylbutanenitrile (21.4%), isobutyronitrile (15.4%), 2-butyl isothiocyanate (18.1%), and 3-methylbutanenitrile (8.2%), which are autolysis products of amino acid derived glucosinolates (GSL). Isopropyl GSL (Glucoputranjivin) upon activation of the glucosinolate-myrosinase system may yield isopropyl isothiocyanate while 2-butyl GSL may give rise to 2-butyl isothiocyanate [31]. Their theoretical autolysis is illustrated in Figure 5.

Isothiocyanates (ITCs) correspond to 50.0% of CC essential oil while nitriles correspond to 47.4% of the total oil. The pharmacological potential of isothiocyanates and nitrile containing compounds has been widely demonstrated in the literature. The anticarcinogenic effects of isothiocyanates (ITC) have been extensively studied in animal models with approximately 20 natural and synthetic isothiocyanates inhibiting chemically-induced carcinogenesis. Chemoprotection is consequently conferred on a variety of target organs, which include the mammary gland, liver, lungs, forestomach, oesophagus, small intestine, bladder, and colon [32].

Experimental evidence has been provided to support the inhibitory effects of phenylethyl ITC (PEITC), benzyl ITC (BITC), and phenyl ITC (PITC) on lung tumorigenesis and 6-*O*-methylguanine formation (DNA-adduct formation in the 4-(methylnitrosamino)-1-(3-pyridyl)-1-butanone (NNK)-induced tumors) present in the lung cell DNA of A/J mice treated with nicotine-derived nitrosamine ketone (NNK) [33,34,35]. NNK accounts for one of the most potent tobacco carcinogens capable of inducing lung tumors in smokers [36]. Similarly, BITC was also shown to inhibit 7,12-dimethylbenz[a]-anthracene (DMBA)-induced mammary tumor formation in female Sprague–Dawley rats [37].

The mechanism of anticarcinogenic activity of ITCs has been attributed to potent competitive and irreversible (depending on conditions) inhibition of carcinogens, Cytochrome P-450 (CYP), enzymes necessary for metabolic processing and subsequent activation of carcinogens. An example is seen in the blunting of catalytic activity of CYP enzymes, including CYP1A1, 1A2, 2B1/2, 2E1, and 3A4 by Sulforaphane (SFN) [38]. Isothiocyanates can also induce phase II enzymes such as quinone reductase (QR) and Glutathione S-transferase (GST) activity in rodent tissue detoxification enzymes. Phase II enzymes catalyse conjugation reactions, which facilitate biotransformation of xenobiotics endo biotics and carcinogen metabolism such as GST. However certain phase II enzymes have been characterised as agents capable of catalysing phase I reactions with no ties to biotransformation (QR-type enzymes). Isothiocyanates achieve phase II enzyme induction by stimulating transcription of phase II enzymes via the ubiquitous AP-1-like enhancer element found in the upstream regulatory region of some GST and QR genes [39].

Historically ITCs have been shown to exhibit biocidal outcomes on a limited number of bacterial pathogens. Aromatic ITCs such as BITC have been reported to have greater antimicrobial capacity for certain Gram-positive bacteria (e.g., *Bacillus cereus*, *Bacillus subtilis*, *Listeria monocytogenes*, and *Staphylococcus aureus*) and Gram-negative bacteria (e.g., *Aeromonas hydrophila*, *Pseudomonas aeruginosa*, *Salmonella choleraesuis*, *Salmonella enterica*, *Serratia marcescens*, *Shigella sonnei*, and *Vibrio parahaemolyticus*) in comparison with aliphatic ITCs [40]. 

Nitrile containing pharmaceuticals are widely prescribed for a variety of indications ranging from diabetes to cancer, with additional compounds undergoing clinical development [41]. The role of the CN unit as a hydrogen bond acceptor has been extensively studied in medically active nitriles [42]. Presence of an adjacent alkyl group (to the nitrile group) is primarily responsible for toxicity due to the accumulation of cyanogenic glycosides. Alkylnitriles may undergo oxidation in the liver to cyanohydrins yielding cyanide release [43]. Benzyl nitrile has been identified as an important precursor for the synthesis of various pharmacological product examples of which include analgesics such as Pethidine, antimalarials such as pyrimethamine, antidepressants such as Venlafaxine, stimulants such as Methylphenidate, hypnotics such as Phenobarbital, antihistamines such as Chlorphenamine, antitussives such as isoaminile and oxeladin etc. [44,45].

Other minor compounds present in the oil are also worth some pharmacological consideration. α-Terpineol, a volatile monoterpenoid, has a vast number of commercial uses in perfumery and cosmetics as well as being characterised as having anticonvulsant and anticancer properties. This compound was also effective in controlling chemically (pentylenetetrazole) and physically (maximal electroshock)-induced convulsions in animal models up to 200 mg/kg doses, with complete anticonvulsant protection being observed during the maximal electroshock test [46].

Evidence has also been put forward to support the anticancer potential of α-terpineol via NF-kB pathway inhibition. This may suggest that α-terpineol will inhibit tumor cell growth by this mechanism [47]. Linalool an acyclic monoterpene alcohol is commercially utilised as a fragrance in the majority of household cleaning products, cosmetics, and perfumery due to its characteristic lavender scent. More recently, it has been assayed for pharmacological effectiveness allowing for its anticonvulsant, sedative, antidepressant, anxiolytic, and analgesic effects to be elucidated [48]. Linalool was also shown to reduce opioid requirements for morbidly obese patients following undergoing laparoscopic adjustable gastric banding compared to a placebo (*p* = 0.004) [49]. Similarly, the therapeutic value of eucalyptol beyond its commercial uses in consumer products has also been extensively illustrated. It has been implicated in the inhibition of polyclonal stimulated cytokine production in vitro, which could suggest its use in the treatment of inflammatory conditions such as asthma and Chronic Obstructive Pulmonary Disease (COPD) [50]. However, these three compounds together account for less than 2.0% of the overall CC oil composition. 

The derived dendrogram of the AHC (Figure 3) highlights two distinct classes of samples (C1 and C2). Class C1 groups four essential oils obtained from the *C. cartilaginea* (CC) sample investigated in the present study, *C. spinosa* L. var. *aravensis* (CA), *Capparis spinosa* var. *mucronifolia* (CA2), and *Capparis ovata* Desf. var. *palaestina* (CP). These samples were characterised by high relative amounts of (a) and (c) compared with other samples analysed. The oil sample obtained from the aerial parts of *C. spinosa* L. var. *aravensis* from Egypt (CA) differs slightly from the other three samples by producing terpenoid compounds, such as camphor (1.66%) and *p*-cymene (2.93%).

Class C2 samples consist of all other analysed essential oil samples, the majority of which were derived from the aerial parts of the plant except for the sample CL7 which was derived solely from seeds. These oil samples obtained from different parts of the same species were in all cases recognised as statistically similar and attributed the lowest Euclidian distance (e.g., *Capparis spinosa* L. samples CL1, CL11a-c, CT). Even though the composition of essential oils was not identical for different plant organs from one population of the same species, the plant organ specification did not significantly affect the MVA result in this case.

Furthermore, based on the results of the AHC, one could speculate the composition of essential oils from geographically analogous locations are statistically similar. For example, the essential oil derived from *C. spinosa* leaves from Iran (CM) and Syria (CL10) were similar, characterised by having high relative amounts of thymol. Both locations are known to experience a Mediterranean climate as a result of patterns of average precipitation, temperature, and natural vegetation. They are classed as a Csa-type climate according to the Köppen–Geiger Climate Classification system indicating that the temperature of the warmest month would be 22 °C [51]. Similarly, Class C1 samples all show significant component similarities as that climate in Saudi Arabia, Egypt, and Jordan is determined by vegetative dryness/aridity (Bwh-type climate). The literature has reported susceptibility of secondary metabolites found in capers to environmental factors such as light, temperature, moisture, pressure, or altitude. These environmental stressors may facilitate genetic changes or biotransformation epigenetically by methylation or acetylation [1]. Hence, we may infer that environmental stressors can impact the nature, and, to a lesser degree, the amounts of constituents produced by members of the genus *Capparis*.

The quadrant grouping display of the PCA (Figure 4) reports similar information as has been deduced from the AHC. Sample CC is depicted in the upper right quadrant isolated from all other analysed samples except for the two species from Jordan (CA2 and CP). Similar negative F1 and F2 values were reported for CL1, CT, CL11a-c, and CL7, which suggested that these are closely related species. Their depiction in the bottom left quadrant of the plot can be attributed to the absence of components associated with the principal component such as the nitrile containing compounds and isothiocyanates. Similarly, samples CM and CL10 have been sequestered slightly to the upper left quadrant, indicating that these samples contain constituents such as thymol and hexenal that contribute slightly to the estimation of the second principal component (F2).

## 4. Materials and Methods

### 4.1. Plant Material

Samples of CC leaves were collected from the Al-Taif region Saudi Arabia in June 2019. The plant material was identified by Prof. Ammar Bader, voucher specimens (SA-UK 2019-2) were deposited in the herbarium of the pharmacognosy lab, Umm Al-Qura University. The collected leaves were air-dried to obtain a stable weight and finely ground.

### 4.2. Extraction of the Essential Oil

Air-dried plant material of CC (two 100-g batches) underwent hydrodistillation with 500 mL of deionised H_2_O per batch for 3 hours using a Clevenger-type apparatus. Once the process was completed the essential oil was clearly separated out on top of the immersing medium (water). The plant water and essential oil were carefully collected via the aperture at the bottom of the apparatus. The oil yield (0.4 mL) was determined by sight using the graduation of the apparatus. The distillates were stored at low temperature in sealed vials until analysis.

### 4.3. GC–MS and GC–FID Analyses

The GC–MS analysis was performed in triplicate for both samples using a Hewlett-Packard 6890N gas chromatograph coupled with a 5975B mass selective detector (MSD; Agilent Technologies, Santa Clara, CA, USA) operating at 70 eV over a mass range of 35–500 amu and a scanning speed of 0.34 and equipped with a DB-5MS fused-silica capillary column (5% phenylmethylsiloxane; length—30 m, internal diameter—0.25 mm, film thickness—0.25 mm). The oven temperature was raised from 70 °C to 290 °C at a heating rate of 5 °C/min and held isothermally for 10 min; injector temperature, 250 °C; interface temperature, 300 °C; carrier gas, He (1.0 mL/min). Each ml of sample oil was dissolved in Et_2_O in the ratio 1:1000 and injected in a pulsed split mode. Flow rate was 1.5 mL/min for the first 30 s (0.50 min) then modified to 1.0 mL/min for the reminder of the run; split ratio 40:1. The GC (FID) studies were performed under the identical experimental circumstances as the GC–MS analyses, using the same column. The percentage composition was calculated without the use of correction factors from the GC peak areas.

### 4.4. Compound Identification

The essential oil components were identified on the basis of (i) their linear retention in- dices (RI) determined experimentally relative to the Rt of n-alkanes (C_8_–C_40_) on the DB-5MS column in comparison to and NIST Standard Reference Database 69: NIST Chemistry WebBook; (ii) mass spectra (MS) data compared against commercially available MS libraries Wiley 6, NIST02 and NIST 17.

### 4.5. Multivariate Statistical Analyses (MVA)

Essential oil components of 20 samples of *Capparis* (including present study) were mutually analysed by means of MVA, specifically principal component analysis (PCA) and agglomerative hierarchical clustering (AHC). The MVA was carried out using the Excel program plug-in XLSTAT version 2021.1.1. The mean values of the correlative % content for each constituent of the compared essential oils were utilised as variables (total number: 10). It should be noted that only components with content greater than 1% in at least one sample were considered for the purpose of the MVA. The agglomerative hierarchical cluster was generated using the Pearson dissimilarity measure. In this case the aggregation criteria included unweighted pair group average, simple linkage, complete linkage, and Euclidean distance factored against weighted pair-group average, unweighted pair-group average, and Ward’s method as aggregation criteria. A Pearson (n)-type PCA was computed.

## 5. Conclusions

The results of the PCA indicate that CC essential oil is not similar with respect to its chemical constituents to any of the analysed species within the genus. Although CC produces isopropyl isothiocyanate to a considerable degree as seen in other species, this factor is not sufficient to show statistical similarity.

Furthermore, the results of the statistical analyses distinctively suggest that environmental stressors can be implicated in the essential oil profile of *Capparis* species as samples obtained from contrasting geographical climates show significant component dissimilarities. The opposite is seen for geographically analogous plant oil samples; geographically analogous samples appear to produce the same constituents to nearly the same degree.

## Figures and Tables

**Table 1:** Composition of the leaf essential oil of *Capparis cartilaginea* from Saudi Arabia.

Compounds	Peak Area%	Retention Time	Retention Index ^a^	Class	Identification ^b^
Isobutyronitrile (d)	15.4	2.124	625	Nitriles	RI, MS
(*Z*)-2-Methyl-3-butenenitrile (j)	0.5	2.371	705	Nitriles	RI, MS
2-Methylbutanenitrile (b)	21.4	2.491	717	Nitriles	RI, MS
3-Methylbutanenitrile (e)	8.2	2.528	731	Nitriles	RI, MS
(*E*)-2-Methyl-3-butenenitrile	tr ^c^	2.603	741	Nitriles	RI, MS
Isopropyl isothiocyanate (a)	31.0	3.321	837	Sulphur containing compounds	RI, MS
(*E*)-2-Hexenal	tr	3.523	837	Other	RI, MS
Methyl isopropylcarbamate	tr	3.798	846	Other	RI, MS
(*E*)-1-Isothiocyanato-2-butene (m)	0.2	4.167	872	Sulphur containing compounds	RI, MS
2-Butyl isothiocyanate (c)	18.1	4.571	909	Sulphur containing compounds	RI, MS
Isobutyl isothiocyanate (h)	0.7	4.937	931	Sulphur containing compounds	RI, MS
Benzaldehyde	tr	5.169	952	Other	RI, MS
6-Methyl-5-hepten-2-one	tr	5.440	981	Other	RI, MS
Myrcene	tr	5.496	988	Monoterpene	RI, MS
*p*-Cymene	tr	6.343	1020	Monoterpene	RI, MS
Limonene	tr	6.461	1024	Monoterpene	RI, MS
Eucalyptol (g)	0.8	6.554	1026	Monoterpene	RI, MS
γ-Terpinene	tr	7.064	1054	Monoterpene	RI, MS
*cis*-Linalool oxide (furanoid)	tr	7.339	1067	Monoterpene	RI, MS
*trans*-Linalool oxide (furanoid)	tr	7.706	1084	Monoterpene	RI, MS
Linalool (i)	0.6	7.938	1095	Monoterpene	RI, MS
Nonanal	tr	8.050	1100	Other	RI, MS
Benzyl cyanide (f)	1.9	8.918	1124	Nitriles	RI, MS
Camphor	tr	9.281	1141	Monoterpene	RI, MS
Terpinen-4-ol	tr	10.066	1174	Monoterpene	RI, MS
*p*-Cymen-9-ol	tr	10.167	1186	Monoterpene	RI, MS
α-Terpineol	tr	10.407	1204	Monoterpene	RI, MS
*O*-Methylthymol	tr	11.474	1232	Monoterpene	RI, MS
Cumin aldehyde	tr	11.644	1238	Monoterpene	RI, MS
Piperitone	tr	11.949	1249	Monoterpene	RI, MS
Vitispirane A	tr	12.651	1281	Other	RI, MS
Dihydroedulane IIA	tr	12.883	1289	Other	RI, MS
Thymol	tr	13.010	1291	Monoterpene	RI, MS
Theaspirane A	tr	13.552	1319	Other	RI, MS
α-Terpinyl acetate (k)	0.5	14.267	1346	Monoterpene	RI, MS
Hydroxydihydroedulan (n)	0.3	16.744	1453	Other	RI, MS
β-(*E*)-Ionone	tr	17.611	1486	Other	RI, MS
Dodecanoic acid	tr	19.426	1565	Other	RI, MS
(*Z*)-3-Hexen-1-yl benzoate (l)	0.3	19.789	1565	Other	RI, MS
Cyclooctasulfur	tr	30.110	2014	Sulphur containing compound	RI, MS

^a^ RI: Retention index experimentally determined on a DB-5MS column relative to the Rt of n-alkanes (C_8_–C_40_); the compounds are listed in the order of elution. ^b^ Compound identification: RI and mass spectra mass spectra (MS) data compared against commercially available MS libraries Wiley 6, NIST02 and NIST 17. ^c^ tr: Trace (<0.05%).

## Data Availability

The data presented in this study are available on request from the corresponding author.

## Supplementary Materials

The following supporting information can be downloaded at: https://www.mdpi.com/article/10.3390/plants11192518/s1, Figure S1: TIC Chromatogram of CC essential oil.

## Sample Availability

Samples of the leaves and essential oil are available from the authors.
