# Supplementary figures and images for: Volatiles of Capparis cartilaginea Decne. from Saudi Arabia
Source: Plants (Basel). 2022 Sep 26;11(19):2518. doi: 10.3390/plants11192518 (PMC9572899; doi:10.3390/plants11192518)

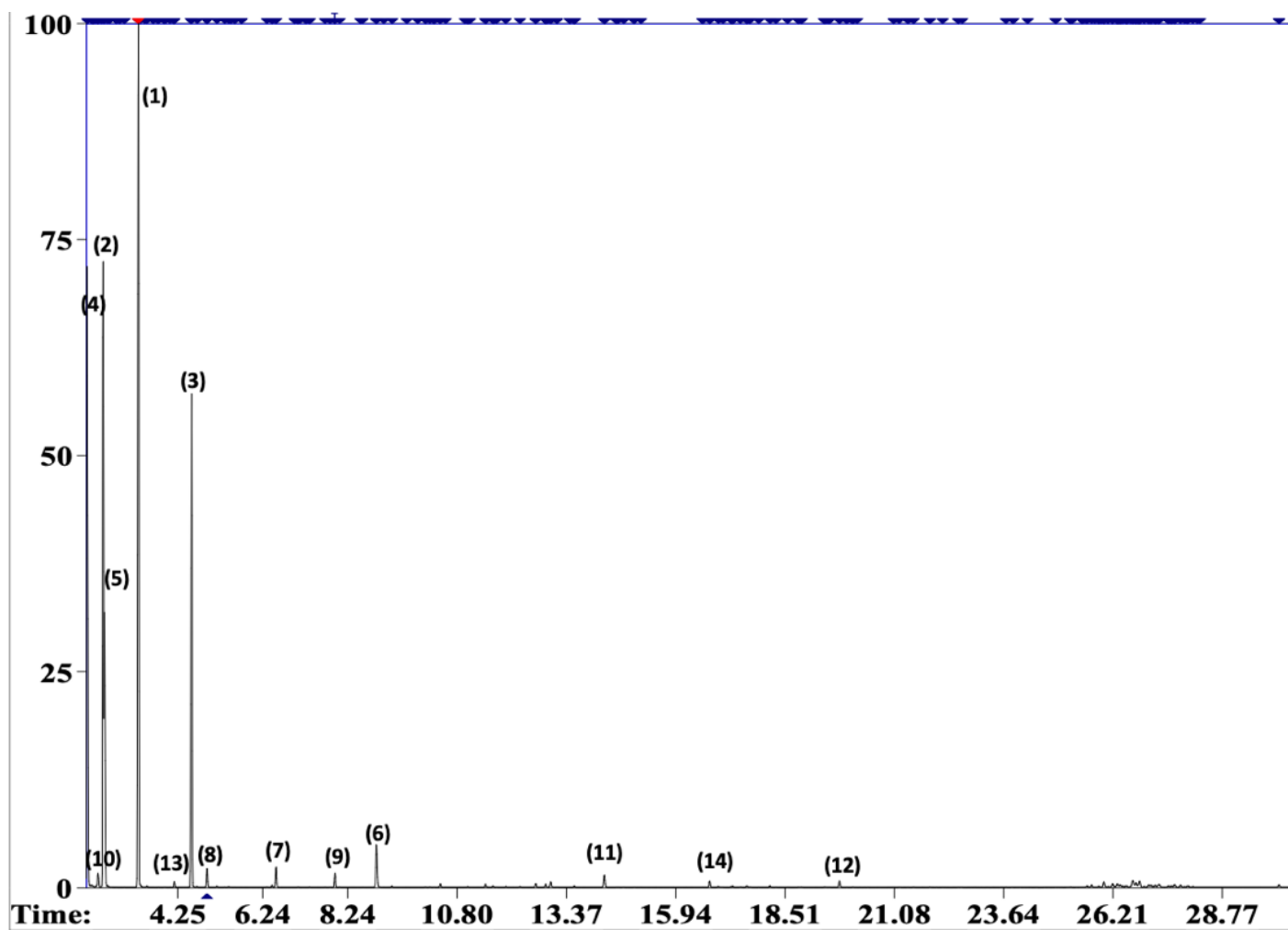

Figure S1. TIC Chromatogram of CC essential oil.

Supplement: Supplementary file 1 [file plants-11-02518-s001.zip › plants-1899417-supplementary.pdf]
